# Mutual impact of adipocytes and colorectal cancer cells growing in co-culture conditions

**DOI:** 10.1186/s12964-023-01155-8

**Published:** 2023-06-14

**Authors:** Joanna Olszańska, Katarzyna Pietraszek-Gremplewicz, Mikołaj Domagalski, Dorota Nowak

**Affiliations:** Department of Cell Pathology, Faculty of Biotechnology, University of Wroclaw, Joliot-Curie 14a, 50-383 Wroclaw, Poland

**Keywords:** Cancer-associated adipocytes (CAAs), Colorectal cancer (CRC), Tumor microenvironment (TME), Adipokines, Lipid droplets, Metabolic reprogramming

## Abstract

**Background:**

Colorectal cancer (CRC) is the third most common malignancy worldwide. CRC cells are situated in an adipocyte-rich microenvironment, which leads to interactions between adipocytes and CRC cells. Upon exposure to cancer cells, adipocytes transform into cancer-associated adipocytes (CAAs), and as a result, they gain features that promote tumor progression. The aim of this research was to shed more light on the detailed role of interactions between adipocytes and CRC cells associated with cancer progression in the context of these alterations.

**Methods:**

To implement adipocyte-CRC cell interaction, a co-culture model was applied. The analyses mainly focused on the metabolic modifications within CAAs and CRC cells, as well as the proliferation and migration potential of CRC cells. The impact of CRC on adipocytes was investigated by qRT-PCR analysis and Oil Red O staining. Proliferation and migration of CRC cells upon co-culture were tested with videomicroscopy, XTT, and a wound healing assay. Metabolic changes within CAAs and CRC cells were investigated based on lipid droplet formation, cell cycle analysis, gene and protein expression by qRT-PCR, and western blotting techniques.

**Results:**

CRC cells induced reprogramming of adipocytes into CAAs, which was connected with downregulation of lipid droplet formation in CAAs and alteration in adipocyte features. CAAs showed decreased metabolism-related gene expression, phosphorylation of Akt, ERK kinases, STAT3, and lactate secretion in comparison to the control. CAAs also promoted the migration, proliferation, and lipid droplet accumulation of CRC cells. After co-culturing with adipocytes, there was a shift to the G2/M phase of the cell cycle according to the differences in cyclin expression.

**Conclusion:**

There are complex bidirectional interactions between adipocytes and CRC cells that may be connected with the induction of CRC cell progression.

Video Abstract

**Supplementary Information:**

The online version contains supplementary material available at 10.1186/s12964-023-01155-8.

## Background

Colorectal cancer (CRC) is the third most common malignancy worldwide [1]. CRC incidences have risen, which has been attributed to increased exposure to risk factors such as poor eating habits and lack of exercise, which lead to obesity [2]. The tumor microenvironment (TME) is a specialized niche composed of tumor cells and other cellular components, such as cancer-associated fibroblasts, adipocytes, and immune cells, as well as extracellular matrix (ECM) elements [3]. Adipose tissue (AT) is the predominant component of TME of colorectal cancer and may increase the metastatic potential and tumor progression [4].

AT is a highly complex and heterogeneous tissue composed of numerous types of cells, such as adipocytes, pericytes, immune cells, endothelial cells, and pluripotent stem cells [4]. Adipocytes are the primary cellular component of this tissue and are lipid-rich cells that mainly store long-chain fatty acids in the form of lipid droplets [5]. Due to the production and secretion of a wide array of effectors, including exosomes, lipids, and adipokines, AT can have paracrine and endocrine effects that control metabolic homeostasis [6]. Aside from their physiological functions, these secreted factors may play a crucial role in cancer proliferation, invasion, and resistance to therapies [7] and may be involved in the initiation and progression of CRC [8].

Although adipocytes may stimulate tumor growth via endocrine effects [6], it has been observed that tumors not surrounded by AT are not associated with obesity. This may suggest that cancer cells are fueled by direct local exposure to proximal fat cells [9]. Tight and long-standing contact between cancer cells and adipocytes was demonstrated to induce the reprogramming of adipocytes into cancer-associated adipocytes (CAAs). Their presence has been observed in various malignancies, including colon, breast, ovarian, prostate, and pancreatic cancers [7].

Cancer-associated adipocytes detected at the invasive front of a tumor exhibit a modified fibroblast-like phenotype upon exposure to cancer cells [10]. In this situation, the number and size of adipocytes decrease, lipid droplets become smaller and dispersed, and triglyceride content decreases. CAAs also indicate a lower expression of genes related to adipocyte differentiation, such as peroxisome proliferator-activated receptor γ (PPARγ), CCAAT/enhancer-binding protein α (c/EBPα), fatty acid binding protein 4 (FABP4), and hormone-sensitive lipase (LIPE). As a consequence of delipidation and changes in fat storage capacity [7], the metabolism of CAAs is switched toward catabolic processes, leading to the release of high‑energy metabolites, including free fatty acids (FFAs), lactate, pyruvate, and ketone bodies [11].

Adipocytes’ contribution to tumorigenesis involves crosstalk between these cells and cancer since the reprogramming of the adipocytes into CAAs occurs as a consequence of the impact of cancer cells [7]. Additionally, cancer cells may promote lipolysis in neighboring adipocytes, take in the released FFAs through specific transporters (e.g. CD36, FABP), and store them in lipid droplets. Cancer cells may also modify their metabolism toward fatty acid oxidation, which is crucial when the demand for ATP is high [5].

Numerous studies have focused on the tumor microenvironment in recent years, but there is still a limited amount of data about the relation between adipocytes and colorectal cancer cells. Given that CRC is located in an AT-rich environment and obesity is a risk factor for this malignancy, it seems extremely important to study the molecular interaction between adipocytes and colorectal cancer cells. Thus, our research focused on understanding the relationship between these cell types.

## Methods

### Cell culture

The human colorectal carcinoma cell lines LS180 (Deutsche Krebsforschungzentrum, Heidelberg, Germany), HCT116, and LoVo (European Collection of Cell Cultures) were cultivated in MEM-α, McCoy′s 5A, and Ham’s Nutrient Mixture F12 (all from Sigma), respectively. Media were supplemented with 10% fetal bovine serum (FBS, Gibco), 2 mM glutamine (Sigma), and antibiotics (10.000 U/ml penicillin, 10 mg/ml streptomycin, 25 µg/ml amphotericin B) (Gibco). Mouse preadipocyte 3T3-L1 cells (ATCC) were cultured in DMEM medium containing 4.5 g/l glucose and 1.5 g/l NaHCO_3_ (IITD PAN, Wroclaw, Poland) supplemented with 10% bovine calf serum (BCS) (Thermo Fisher Scientific), 2 mM glutamine, and antibiotics (10 000 U/ml penicillin, 10 mg/ml streptomycin, 25 µg/ml amphotericin B). All cells were grown at 37 °C in 5% CO_2_ in a humidified atmosphere and passaged using 0.25% trypsin/0.05% EDTA solution (IITD PAN, Wroclaw, Poland).

### Adipocyte differentiation

Two days after reaching around 100% confluence, 3T3-L1 cell differentiation was induced by changing the medium to DMEM 4.5 g/l glucose with 10% FBS, antibiotics (10 000 U/ml penicillin, 10 mg/ml streptomycin, 25 µg/ml amphotericin B) (Gibco), 2 mM glutamine, 0.5 mM IBMX, 0.25 µM dexamethasone, 1 µg/ml insulin, and 2 µM rosiglitazone. After 48 h, the medium was replaced with DMEM 4.5 g/l glucose with 10% FBS, antibiotics (10 000 U/ml penicillin, 10 mg/ml streptomycin, 25 µg/ml amphotericin B) (Gibco), 2 mM glutamine, and 1 µg/ml insulin for next 2 days. Subsequently, the medium was changed to DMEM containing 10% FBS and 2 mM antibiotics and glutamine for the next 7 days. The adipocytes reached maturity after this time [12].

### Co-culture conditions

CAAs were obtained from mature adipocytes by co-culturing them with CRC cells using Transwell inserts (0.4 μm pores, Falcon). Cancer cells were seeded onto an insert (450,000 cells) and on the bottom of a 6-well plate (500,000 cells; control). On the next day, the inserts with CRC cells were put in a plate with mature adipocytes. Cells were grown in these conditions for 7 days, and the medium was refreshed every 3–4 days. A culture of mature adipocytes was run in parallel as a control. After each change, the replaced culture media (with FBS) were centrifuged for 15 min at 1000 × *g* and frozen at − 80 °C. When necessary, they were used as conditioned media for proliferation and migration assays.

After co-culture, CAAs and CRC cells were collected and used for further experiments. For secreted protein expression analysis, cells were washed three times with PBS, and the culture media were changed to fresh ones without FBS for another 72 h. Next, the media were aspirated, centrifuged for 15 min at 1000 × *g,* and frozen at − 80 °C.

### Proliferation assay

To examine the cancer-cell proliferation rate, a Cell Proliferation Kit II (XTT) (Roche) was used according to the manufacturer’s instructions. Briefly, 5,000 cells grown in mono-culture or co-cultures were seeded in the well of 96-well plates in a 1:1 mixture of culture and conditioned medium (with FBS collected as described in the previous section). The XTT mixture was added in parallel samples at 24 h (time 0, T0), 48 h (T24), and 72 h (T48) after cell seeding. After 3 h of incubation at 37 °C, the absorbance was measured spectrophotometrically at 450 nm with a reference wavelength of 630 nm using an ELISA plate reader (BIO-TEK Instruments, Inc.). The results are presented as a proliferation rate (absorbance value at a given time divided by the value at T0) in comparison to control cells from monoculture. The experiments were performed three times, and each independent experiment consisted of four measurements.

### Spontaneous migration assay

To measure the distance and speed of cell migration, CRC cells from co-culture or mono-culture were seeded on 96-well plates (IncuCyte ImageLock, Sartorius) covered with Matrigel (1 mg/ml) (Corning) and incubated for 1 h at 37 °C. Then, phase-contrast time-lapse images were captured for 24 h with time intervals of 2 h using a 10 × objective in an IncuCyte® Live-Cell Analysis System (Sartorius). Pictures were analyzed with ImageJ software and the Manual Tracking plugin [13]. The distance covered by every cell was measured as the total distance based on the cumulative track lengths. Two independent experiments were carried out, and in each one, at least 25 cells were analyzed.

### 3D wound healing assay

Cancer cells derived from co-culture or mono-culture were seeded into Matrigel-coated (0.5 mg/ml) ImageLock 96-well plates (IncuCyte ImageLock, Sartorius) and incubated for 24 h to reach 100% confluence. Then, standardized scratches were made in all wells simultaneously using Wound Maker™ (Essen Bioscience). Subsequently, cells were covered with an additional layer of Matrigel, on top of which a 1:1 combination of culture and conditioned medium (with FBS collected as described in the “Co-culture conditions” section) was added into each well. Phase-contrast time-lapse images were taken every 2 h for 24 h using an IncuCyte® LiveCell Analysis System using a 10 × objective. Representative results were analyzed using the IncuCyte® Scratch Wound Cell Migration Software Module (Sartorius). The relative wound density represents the increase in the area covered by the cells over time. The experiments were performed three times, and each independent experiment consisted of four repetitions.

### qRT‑PCR analysis

To determine the expression level of certain genes in adipocytes and CRC cells after co-culture, RNA was isolated using an RNA purification kit (EURx) for colon cancer cells or TRI Reagent™ Solution (Invitrogen) for adipocytes according to the manufacturer’s protocol. RNA was used for the reverse transcription reaction (0.5 µg from CRC cells or 1 µg from adipocytes), which was done with a High-Capacity cDNA Reverse Transcription Kit (Applied Biosystems) according to the manufacturer’s instructions. Quantitative PCR was performed using PowerUp™ SYBR™ Green Master Mix. We used primers designed for human and mouse genes for cancer cells and adipocytes, respectively. The results were normalized to the expression of a housekeeping gene (eukaryotic translation elongation factor 2 (EeF2) for adipocytes and hypoxanthine phosphoribosyltransferase 1 (HPRT1) for CRC cells) based on the comparative CT (threshold cycle value) method (ΔCT = 2^- (CT gene of interest − CT housekeeping gene). All primers were obtained from Merck. The experiment was performed at least three times, and the sequence of applied primers is shown in Table 1.

**Table 1:** Sequences of utilized primers

	**Gene**		**Forward Primer 5’-3’**	**Reverse Primer 5’-3’**
	***A*bbreviation**	**Full name of the gene**		
**Adipocyte primers**	***Adiponectin***	-	aatcttgcccagtcatgccg	ccttaggaccaagaagacctgc
	***EeF2***	eukaryotic translation elongation factor 2	gacatcaccaagggtgtgcag	tcagcacactggcataggc
	***FABP4***	fatty acid binding protein	gcttgtctccagtgaaaacttcg	ccagtttgaaggaaatctcggtg
	***LIPE***	lipase E, hormone sensitive lipase	ttacgcacgatgacacagtcg	tgtctctgtgtccaggtcaaaatgg
	***PPARγ***	peroxisome proliferator-activated receptor γ	accaacttcggcctcagctctg	tgtaatcagcaaccattgggtcag
	***Resistin***	-	caagacttcaactccctgtttcc	ggaaaccacgctcacttccc
	***SC4MOL***	sterol-C4-methyl oxidase-like protein	gtgttggcgtgttcagctctg	agatggcttcgtgaactatcaggg
	***Perilipin 2***	-	atccgtgtgtgagatggccg	ggcaacaatctcggacgttgg
	***OSBPL9***	oxysterol-binding protein-like 9	gcgtccatcttccctaccag	acgtggcttggagaagtgag
	***FADS1***	fatty acid desaturase 1	cgccaaacgcgctactttacttg	catcctgacccgcgtagtgg
	***MCT1***	monocarboxylate transporter 1	cctatgcatttcccaaatccatc	gatactgctgataggacctcc
	***NHE1***	sodium–hydrogen antiporter 1	ctcatcgcctcaggagtag	ggtgctgatgacgaaggtc
	***Glut1***	glucose transporter 1	gatcactgcagttcggctataac	cctgccaaagcgattaacaaag
**CRC cell primers**	***HPRT1***	hypoxanthine phosphoribosyltransferase 1	caaactttgctttccctggt	tctggcctgtatccaacacttc
	***GLUT1***	glucose transporter 1	cagcaagaagctgacgggt	cagaaaagatggccactgagagg
	***GLUT3***	glucose transporter 3	atggggacacagaaggtcac	cggaaaatatggccacagaca
	***CCND1***	cyclin D1	ttcaaatgtgtgcagaaggaggtc	agggggatggtctccttcatctta
	***CCNA2***	cyclin A2	ctctacacagtcacgggacaaag	ctgtggtgctttgaggtaggtc
	***CCNB1***	cyclin B1	gacctgtgtcaggctttctctg	ggtattttggtctgactgcttgc
	***CDK1***	cyclin-dependent kinase 1	ggaaaccaggaagcctagcatc	ggatgattcagtgccattttgcc

### Western blotting analysis

Adipocytes and CRC cells grown in co-culture or monoculture, washed with PBS, and lysed with urea buffer (50 mM Tris, pH 7.4, 5% SDS, 8.6% sucrose, 74 mM urea, 1 mM dichlorodiphenyltrichloroethane) supplemented with protease and phosphatase inhibitor cocktails (Sigma). The protein concentration was determined using the standard bicinchoninic acid (BCA) procedure (Thermo Fisher). Samples with an identical quantity of protein (5 μg) were separated by 10% polyacrylamide gel electrophoresis in the presence of sodium dodecyl sulfate (SDS-PAGE according to Laemmli procedure [14]) and transferred onto nitrocellulose membranes (GE Healthcare) as described by Towbin et al. [15].

Target proteins were probed with specific antibodies and detected using the Clarity Western ECL Substrate (Bio-Rad) under ChemiDoc (Bio-Rad). Primary antibodies against pERK, ERK, pAkt, Akt (all from Cell Signalling Technologies), STAT3, pSTAT3 (both from Santa Cruz Biotechnology), GLUT1 (Abcam), and FASN (Santa Cruz Biotechnology) and secondary goat anti-rabbit and goat anti-mouse antibodies conjugated with horseradish peroxidase (Cell Signaling Technologies) were applied. Densitometry analysis was done with ImageLab software (ver. 6.0, Bio-Rad), and the results were normalized to the total protein examined by Ponceau S staining. At least three independent experiments were performed.

### Evaluation of lactate secretion level

Lactate-Glo™ Assay (Promega) was used to assess the level of lactate released by CAAs into the medium. The analyzed media were collected after 24 h as described in the “Co-culture conditions” section. The experiment was carried out according to the manufacturer’s protocol, and luminescence was measured using the GloMax Discover plate reader (Promega). The luminescence value was calculated as the percentage of the value of control adipocytes, which was set as 100%. Experiments were conducted in three biological replications involving two measurements each.

### Quantification of lipid content

To visualize the lipid droplets in cancer cells, Oil Red O (Sigma) staining was performed as previously described [12]. Adipocytes and CAAs seeded onto coverslips were washed with PBS and fixed with 4% paraformaldehyde for 20 min. Next, cells were incubated for 15 min with 60% isopropanol, followed by Oil Red O solution staining. The coverslips were mounted on glass slides using Dako® fluorescent mounting medium (Dako). The lipid droplets were observed using a confocal laser scanning microscope (Leica SP8 with LasX 3.3.0 software). The experiment was performed in three biological replicates.

Spectrophotometric measurement was performed for quantitative analysis of the lipid content in adipocytes. After staining with Oil Red O, cells were incubated with 100% isopropanol for 10 min to dissolve the bounded dye. Next, the absorbance at 450 nm was measured. The results are presented as a percentage of the control value, and the experiment was performed three times.

In CRC cells lipid droplets were visualized by LipidSpot™488 (Biotium) staining. Cells obtained from mono-culture or co-culture were seeded onto coverslips. After 24 h, the cells were fixed with 4% paraformaldehyde, permeabilized with 0.1% Triton X-100, stained with LipidSpot™ Lipid Droplet Stains (Biotium) and phalloidin conjugated with Alexa Fluor® 568 (Invitrogen) to visualize filamentous actin. After a series of PBS washes, coverslips were mounted on glass slides using Dako® medium. Lipid droplets were observed using a high-resolution confocal microscopy system (Leica Stellaris STED platform), and images were acquired with the same settings. The positive staining of lipid droplets was quantified using ImageJ software [16] for at least 40 cells each from three independent experiments.

### Cell cycle analysis

Colon cancer cells from co-culture or mono-culture were trypsinized, fixed in ice-cold 70% ethanol, and stored at − 20 °C for 24 h. Then, cells were washed three times with PBS to remove ethanol fixation, incubated with RNase A (10 μl/ml) (Sigma-Aldrich) for 45 min at room temperature, and stained with propidium iodide (0.25 mg/ml, (Sigma-Aldrich)) for at least 15 min. The DNA content was analyzed by NovoCyte (ACEA Biosciences Inc.), and the cell cycle phase distributions were analyzed using NovoExpress.

### Statistical analysis

All data are shown as the mean ± standard deviation (SD). Significance was determined with GraphPad Prism 7 software and a one-way ANOVA followed by Tukey’s test or a Kruskal–Wallis test. Alternatively, a student’s t test was applied. Significance test was set at *p* < 0.05 (*), *p* < 0.01 (**), *p* < 0.001 (***), or *p* ≤ 0.0001 (****).

## Results

### Impact of CRC cells on adipocytes

Our previous studies demonstrated that one the adipokine apelin stimulated CRC cell movement [17]. To expand on that observation, we aimed to examine the cross-talk between adipocytes and CRC cells instead focusing on the impact of only one adipokine. Moreover, to better explore the interactions between these cells, we used several CRC cell lines: two primary colorectal carcinomas (LS180 and HCT116) and a metastatic cell line (LoVo). As a model to achieve adipocytes, we employed a 3T3-L1 cell line in our experiments. After maturation, these cells showed the morphology and gene expression of white adipose tissue and are one of the most commonly used in vitro models to evaluate adipose tissue biology [18].

**Fig. 1:**
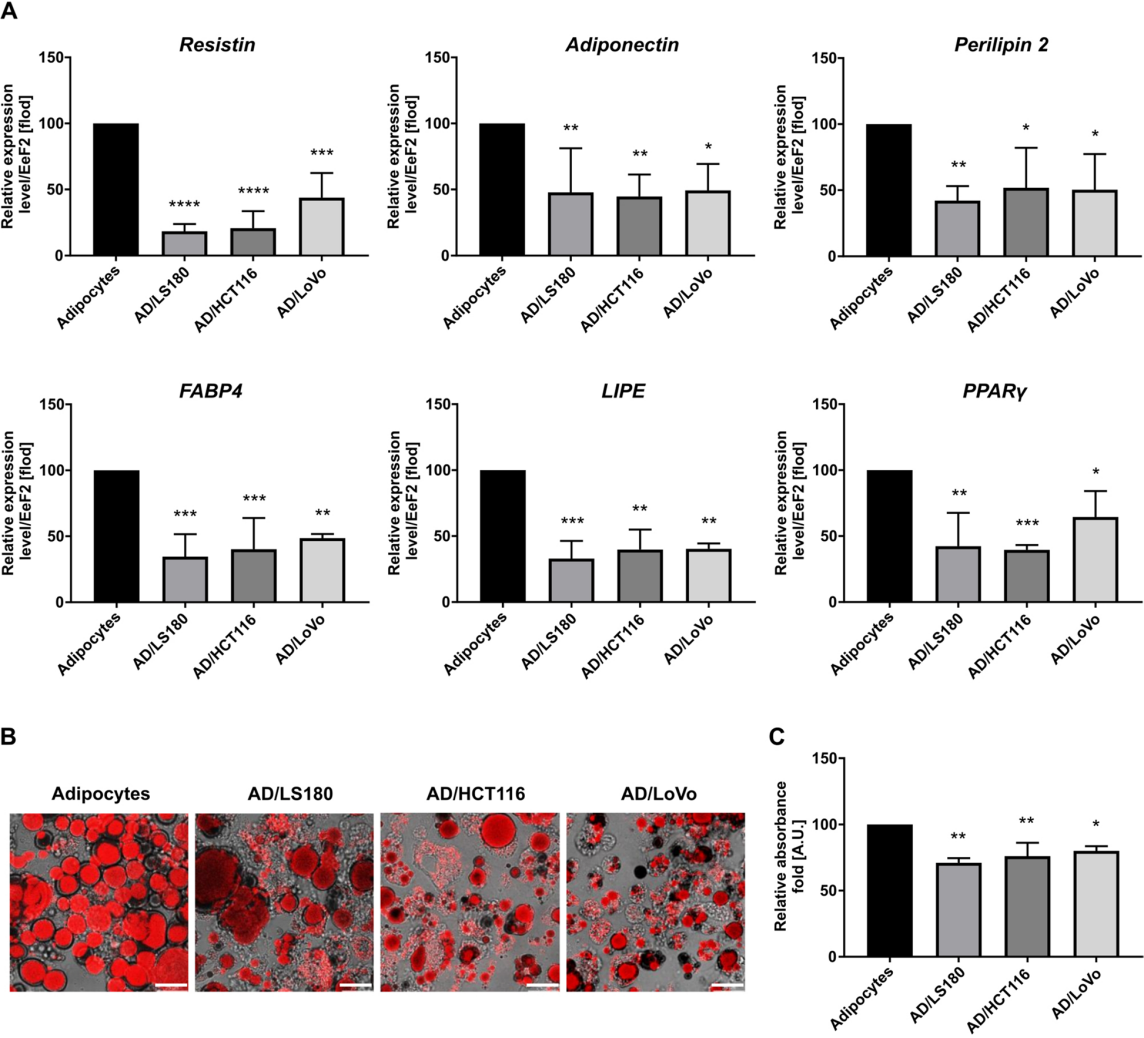
Identification of CAAs. **A** Real-time PCR analysis of genes characteristic of adipocytes: resistin, adiponectin, perilipin 2, FABP4 (fatty acid binding protein 4), LIPE (hormone sensitive lipase), and PPARγ (peroxisome proliferator-activated receptor γ) in control adipocytes and co-cultured with LS180, HCT116, and LoVo cells (AD/LS180, AD/HCT116, and AD/LoVo, respectively). Relative expression was compared to level of EeF2 (eukaryotic translation elongation factor 2) and presented as a percentage of control result. **B** Visualization of lipid droplets (red) in adipocytes and CAAs with Oil Red O staining. Scale bar: 25 µm. **C** Quantitative analysis of lipid content using spectrophotometric measurements calculated as percentage of control result. All results are shown as the mean ± SD of at least three biological repetitions. Asterisks indicate statistical significance in comparison to control adipocytes at *p* ≤ 0.05 (*), *p* ≤ 0.01 (**), *p* ≤ 0.001 (***), *p* ≤ 0.0001 (****)

We have studied the formation of CAAs induced by CRC cells based on the presence of adipocyte differentiation markers. We chose genes that are described in the literature as features of mature adipocytes. Adiponectin and resistin belong to the adipokine family and are secreted by mature adipocytes. FABP4 and LIPE (hormone-sensitive lipase) are markers of adipocyte differentiation [19], just like PPARγ (peroxisome proliferator-activated receptor γ), a key regulator of adipogenesis and adipocyte differentiation, whereas perilipin protects lipid droplets from lipolysis [20]. Real-time PCR analysis showed that expression levels of *LIPE*, *resistin*, *perilipin*, *FABP4*, *adiponectin,* and *PPARγ* were significantly reduced in adipocytes co-cultured with all CRC cells used in comparison to the control consisting of mono-cultured adipocytes (Fig. 1A).

Another hallmark of the adipogenic differentiation of adipocytes is the storage of lipids in lipid droplets. The microscope observations revealed a decline in lipid content in adipocytes under the influence of CRC cells (Fig. 1B), which was also proven by quantitative analysis using spectrophotometric measurement (Fig. 1C). Decreased expression levels of molecules characteristic of differentiated adipocytes and the loss of lipid content confirmed that CRC cells induce the generation of CAAs. Our results also showed that all tested cancer cells similarly contribute to this process.

**Fig. 2:**
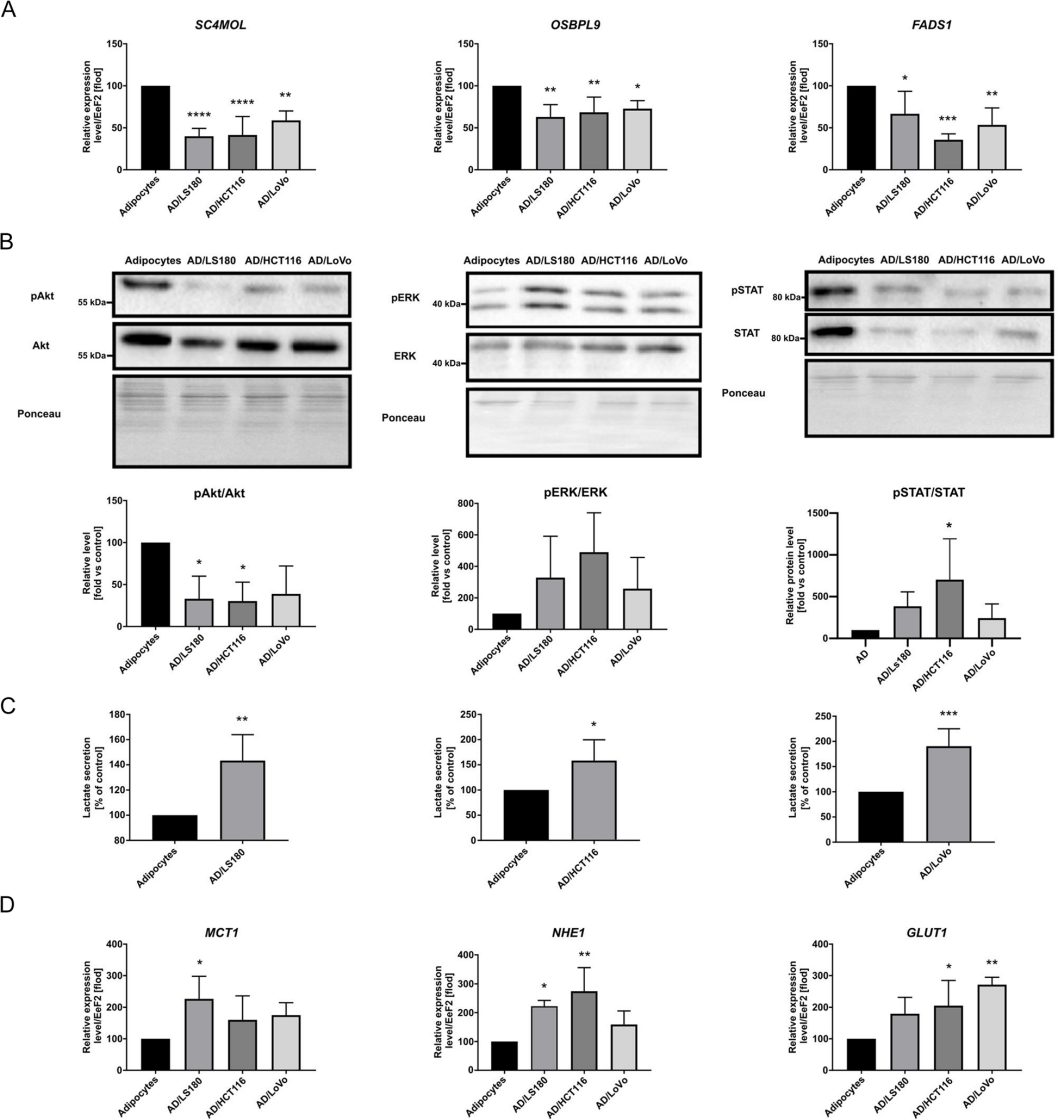
The levels of important molecules for CAAs metabolism. Results of qRT-PCR analysis of (**A**) SC4MOL (sterol-C4-methyl oxidase-like protein), OSBPL9 (oxysterol-binding protein-like 9), FADS1 (fatty acid desaturase 1), (**B**) western blotting analysis of pAkt/Akt, pERK/ERK, and pSTAT3/STAT3 level in cell lysates of control adipocytes or co-cultured with CRC cells. Results were normalized to Ponceau S staining. **C** The level of secreted lactate by control adipocytes and CAAs measured using a chemiluminescent reaction and presented as a percentage of control result. **D** MCT1 (monocarboxylate transporter 1), NHE1 (Na/H + exchanger 1), and Glut1 (glucose transporter 1) expression levels in adipocytes co-cultured with LS180, HCT116, and LoVo cells (AD/LS180, AD/HCT116 and AD/LoVo, respectively) were compared to that of control adipocytes. Relative expression was normalized to EeF2 (eukaryotic translation elongation factor 2) and presented as a percentage of control result. All results are shown as the mean of at least three biological replicates ± SD. Asterisks indicate statistical significance in comparison to control adipocytes at *p* ≤ 0.05 (*), *p* ≤ 0.01 (**), *p* ≤ 0.001 (***), and *p* ≤ 0.0001 (****)

Since we detected delipidation of adipocytes co-cultured with CRC cells, we next explored the possible reasons for the observed phenomenon. Quantitative RT-PCR analysis showed that CAAs had significantly decreased levels of molecules employed in lipid metabolism: *SC4MOL* (*sterol-C4-methyl oxidase-like protein*), *OSBPL9* (*oxysterol-binding protein-like 9*), and *FADS1* (*fatty acid desaturase 1*) (Fig. 2A). SC4MOL is involved in the post-squalene synthesis pathway and catalyzes the first step in the demethylation of 4,4′-dimethyl sterols [21]. OSBPL9 belongs to a group of proteins that mediates oxysterol metabolism and bioactivity in cells [22]. FADS1 is a key rate-limiting enzyme of polyunsaturated fatty acids and catalyzes dihomo-gamma-linolenic acid to arachidonic acid [23].

Additionally, we analyzed the activation of intracellular signaling pathways participating in the processes of adipogenesis and lipolysis. As shown in Fig. 2B, phosphorylation of Akt (protein kinase B, MW 60 kDa) was substantially decreased in adipocytes co-cultured with LS180 and HCT116 cells, which was also observed as a tendency in the case of adipocytes co-cultured with LoVo cells. This protein promotes preadipocyte differentiation [24]. We also found elevated levels of phosphorylated ERK (extracellular signal-regulated kinase, MW 44/42 kDa) and STAT3 (signal transducer and activator of transcription 3, MW is 91 kDa) (Fig. 2B), which may participate in the lipolysis process [25, 26]. However, this alteration can be better described as a trend (except for pSTAT3/STAT3 in HCT116/AD).

Lactate released by the microenvironment cells may be a source of energy for cancer cells and might lead to tumor niche acidification, which stimulates cancer progression. Therefore, we estimated the level of this metabolite secreted by adipocytes and CAAs. The results presented in Fig. 2C indicate that adipocytes co-cultured with CRC cells released a higher level of lactate. Furthermore, we analyzed the expression of ion and glucose-metabolite transporters. The results showed a higher level of mRNA of *monocarboxylate transporter 1* (*MCT1*) in AD/LS180, and *Na/H*^+^ *exchanger 1 (NHE1)* in CAAs compared to control cells (this was observed as a tendency for AD/LoVo) (Fig. 2D)*.* The molecules cooperate together to maintain intracellular pH balance. MCT1 transports lactate together with H^+^ ions in the same direction, and NHE1 pumps accumulated protons outside the cells [27]. In addition, we detected a higher expression level of *glucose transporter 1* (*Glut1*) in AD/HCT116 and AD/LoVo cells (Fig. 2D).

### Alterations in CRC cell proliferation, migration, and metabolism upon co-culture with adipocytes

**Fig. 3:**
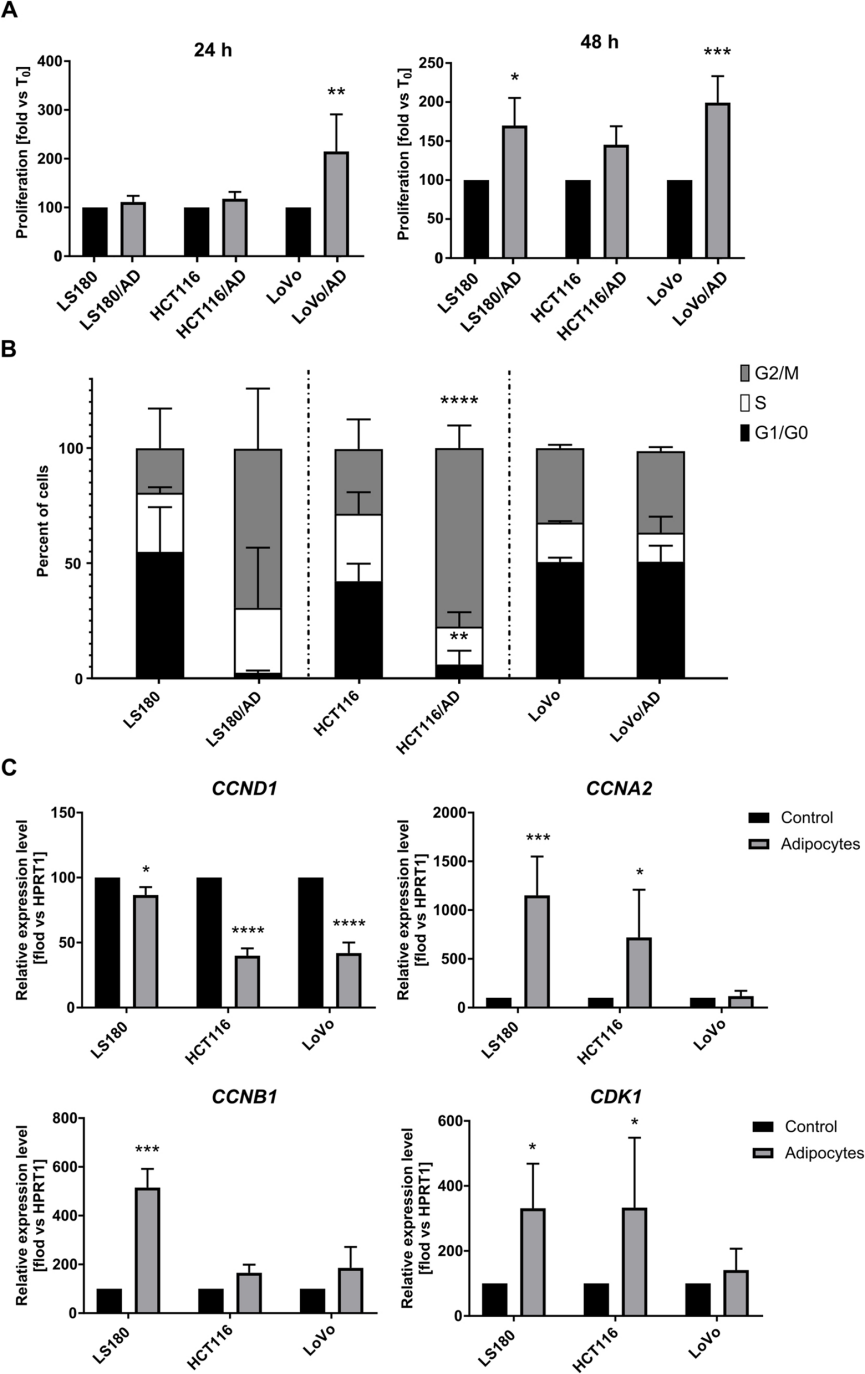
Proliferation abilities of CRC cells co-cultured with adipocytes. **A** The proliferation level was measured using XTT assay after 24 h and 48 h. Results are presented for cells co-cultured with adipocytes (LS180/AD, HCT116/AD, and LoVo/AD) as a percentage of control (LS180, HCT116, and LoVo cells, respectively). **B** Results of cell cycle analysis using flow cytometry technique. The graph shows the distribution of cells in the different phases of the cell cycle in comparison to the control. **C** The relative expression level of CCND1 (cyclin D1), cyclin A2 (CCNA2), cyclin B1 (CCNB1), and CDK1 (cyclin-dependent kinase 1) in CRC cells obtained from co-cultures with adipocytes and compared with mono-cultured cells. Relative expression was normalized to the level of HPRT1 (hypoxanthine phosphoribosyltransferase 1) and presented as a percentage of control. All experiments were conducted three times, and the mean ± SD is shown. Asterisks indicate statistically significant differences between control and cancer cells co-cultured with adipocytes. The significance level was set at *p* ≤ 0.05 (*), *p* ≤ 0.01 (**), *p* ≤ 0.001 (***), or *p* ≤ 0.0001 (****)

Next, we looked at the impact of CAAs on colorectal cancer cells. Published data report that cancer-associated adipocytes can stimulate cell invasiveness in various types of cancer [10]. Thus, we also investigated the effect of adipocytes on the proliferation of colorectal cancer cells using the XTT assay. Co-cultures with adipocytes stimulated the growth of CRC cells in comparison to control cells growing in mono-culture (Fig. 3A). To clarify the mechanism of this observation, we tested the distribution of cell cycle phases in CRC cells cultured with adipocytes. Flow cytometry analysis revealed statistical significance for HCT116/AD and a trend in the case of LS180/AD of an increased percentage of cells in G2/M phases, as well as a decreased number of cells in the G1/G0 phases of the cell cycle (Fig. 3B).

Moreover, we examined the mRNA level of cyclins, which are characteristic of G1, M, and G2 phases. In these stages, we observe differences in the percentage of cells after co-culture with adipocytes in comparison to the control. The qRT-PCR analysis revealed a significant reduction in the transcription level of *cyclin D1 (CCND1)* in all CRC cells cultivated with adipocytes. In addition, we noticed relevant overexpressions of *cyclin A2 (CCNA2), cyclin B1 (CCNB1),* and *cyclin-dependent kinase 1 (CDK1)* in LS180/AD and HCT116/AD (except for *CCNB1*, which had an elevated level in HCT116/AD as a trend) (Fig. 3C).

The CCND1 protein belongs to the G1 cyclin family and regulates the entry of cells into the cell cycle in response to extracellular growth factors or mitogens. Further, CCNA2 represents the S-phase cyclins, and CCNB1 represents M-phase cyclins, which are essential for the initiation of DNA replication and entry into mitosis, respectively [28]. CDK1 binds to cyclin B1 and then controls the G2/M transition and progression through cell division [29]. It is associated with CCNA2 in the regulation of the S-G2 transition and G2 phase progression [30].

**Fig. 4:**
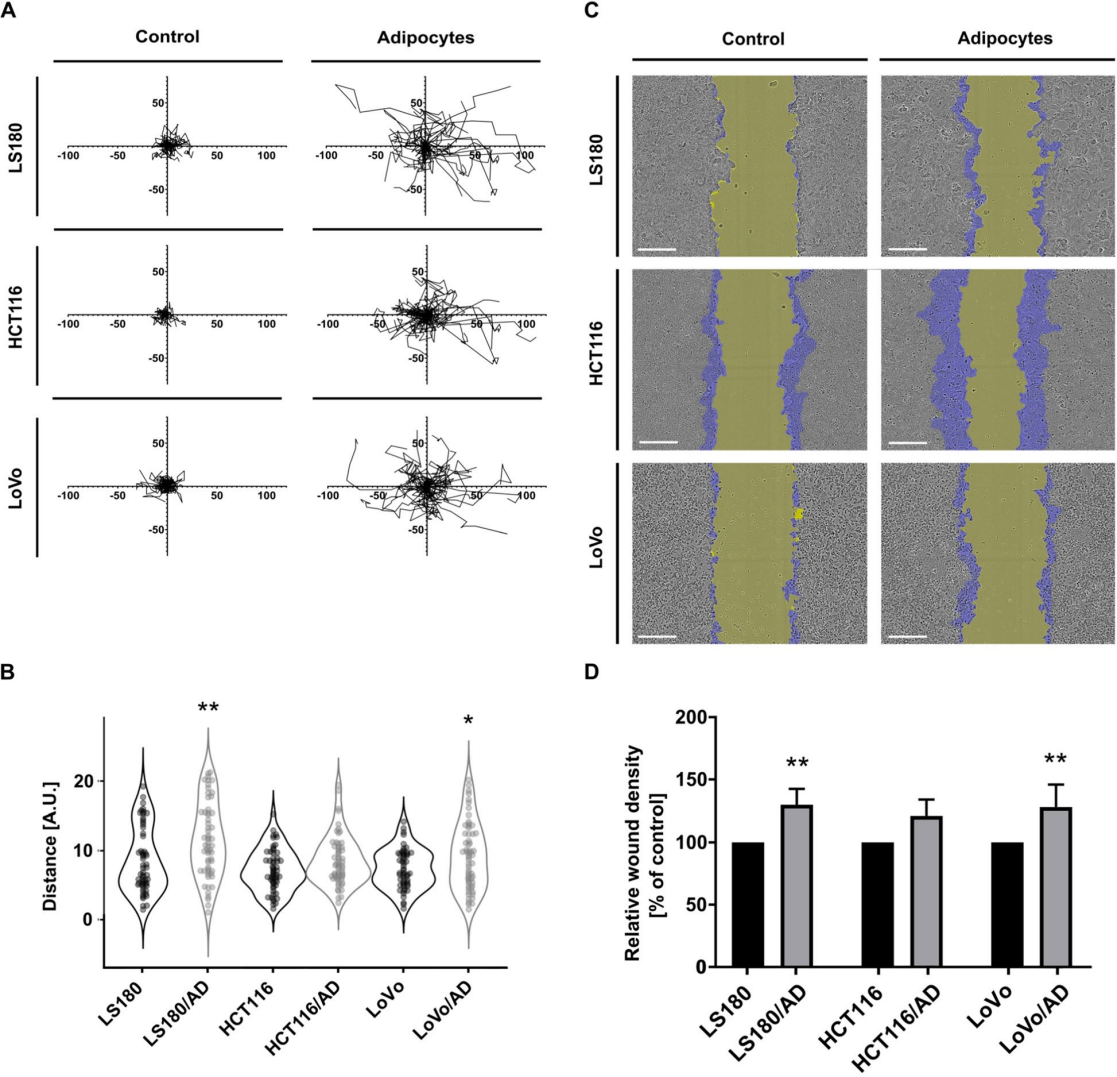
Migration potential of CRC cells after co-culture with adipocytes. **A**, **B** The ability of CRC cells to spontaneously migrate on the Matrigel layer was evaluated using the IncuCyte Live-Cell Imaging System with 2-h intervals. (**A**) Spider graphs show trajectories of migrating cells and (**B**) the chart shows distances covered by cells during 24 h. At least 25 cells were quantified per repetition. **C** Cell migration in 3D wound healing assay. In representative photos, the wound after 24 h is marked in yellow, and the surface on which cells have migrated is shown in violet. Scale bar: 300 µm. **D** Graph of relative wound density determined by IncuCyte Live-Cell Imaging System. All experiments were conducted three times, and the mean ± SD is shown. Asterisks on the graphs indicate statistically significant differences between control and cancer cells co-cultured with adipocytes. The significance levels were set at *p* ≤ 0.05 (*), *p* ≤ 0.01 (**)

We also looked at the impact of adipocytes on the migration potential of CRC cells in 2D conditions, where cells were seeded separately on a Matrigel layer and could move spontaneously without any external stimulation. In this assay, increased migration abilities of CRC cells co-cultured with adipocytes were detected in comparison to mono-cultured cells (control) (with statistical significance for LS180/AD and LoVo/AD and a trend in the case of HCT116/AD) (Fig. 4A and B).

To evaluate the invasive abilities of cancer cells, we also performed a 3D wound healing assay, in which cells were placed between two layers of Matrigel to imitate basement membrane conditions. A covered scratch was visible as a higher blue area in the pictures (Fig. 4C). The relative wound density was elevated in CRC cells co-cultured with adipocytes in comparison to control cells (significantly in LS180/AD and LoVo/AD and as a tendency in HCT116/AD) (Fig. 4D).

**Fig. 5:**
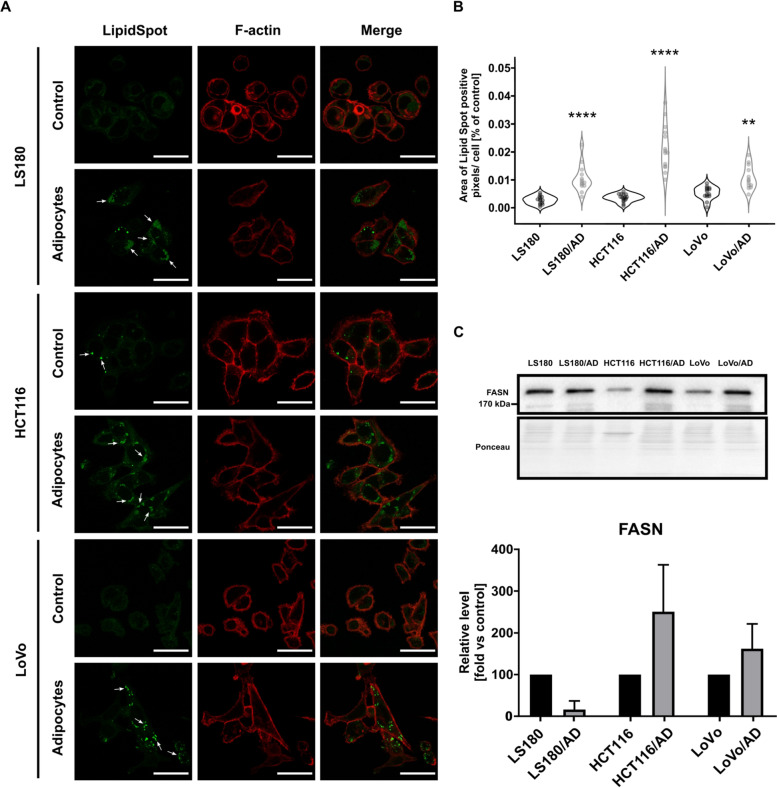
Lipid metabolism of CRC cells co-cultured with adipocytes. **A** Visualization of lipid droplets in control LS180, HCT116, and LoVo cells and co-cultured with adipocytes (LS180/AD, HCT116/AD and LoVo/AD, respectively) with LipidSpot^TM^ staining (green- lipid droplets, red– F-actin – for cells shape indication). Scale bar: 25 µm. **B** Quantification of LipidSpot^TM^ positive signals presented as violin plots. At least 30 cells were quantified per repetition. **C** Western blotting analysis of FASN (fatty acid synthase) level in cell lysates of control LS180, HCT116, and LoVo cells and cells co-cultured with adipocytes (LS180/AD, HCT116/AD, and LoVo/AD, respectively). Results were normalized to Ponceau S staining. Experiments were conducted three times, and the mean ± SD is shown. Asterisks indicate statistically significant differences between control and cancer cells co-cultured with adipocytes. The significance levels were set at *p* ≤ 0.01 (**) and *p* ≤ 0.0001 (****)

Tumor cells have an increased energy requirement due to the rapid proliferation and migration. Energy sources can include high-energy fatty acids, which cancer cells can store in lipid droplets [31]. During microscopic observations, we noticed an accumulation of lipid droplets in CRC cells cultivated with adipocytes (Fig. 5A), which was also confirmed by quantification of a positive signal in the pictures (Fig. 5B). Because lipid droplets could be synthesized de novo in cancer cells, we also evaluated the level one of the key enzymes participating in this process. Elevated expression of fatty acid synthase (FASN) was detected in HCT116 and LoVo cells co-cultured with adipocytes (visible as a tendency) and reduced in LS180 cells (Fig. 5C). The changes in FASN amounts might at least partly explain the elevated content of lipid droplets in CRC cells cultivated with adipocytes.

**Fig. 6:**
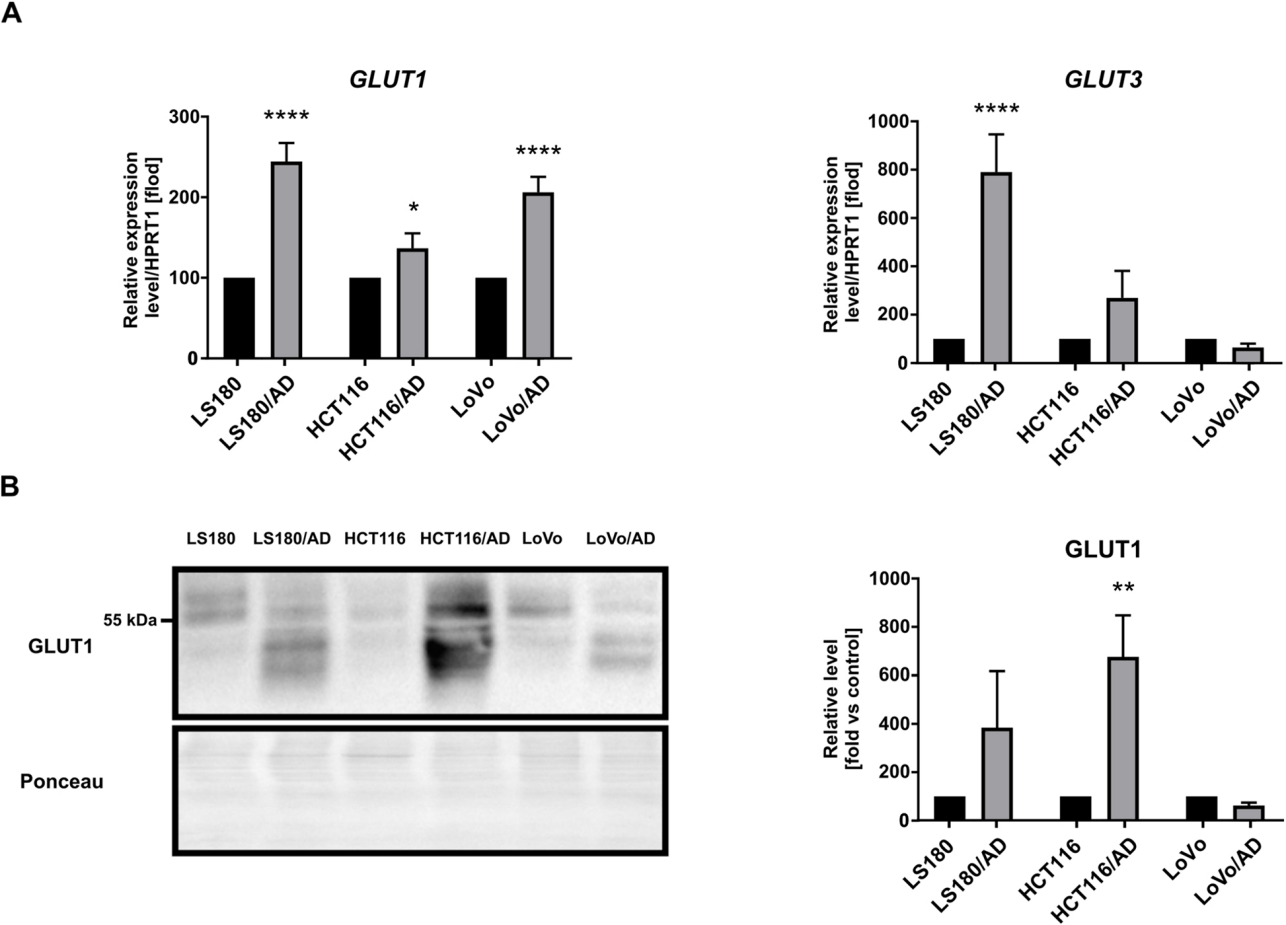
Expression of glucose transporters in CRC cells. **A** qRT-PCR analysis of GLUT1 and GLUT3 expression level in CRC cells of control LS180, HCT116, and LoVo cells and cells co-cultured with adipocytes (LS180/AD, HCT116/AD, and LoVo/AD, respectively). Relative expression was compared to the level of HPRT1 (hypoxanthine phosphoribosyltransferase 1) and presented as a percentage of the control result. All experiments were done three times and the mean ± SD is shown. **B** Western blotting analysis of GLUT1 (glucose transporter-1) level in cell lysates co-cultured with adipocytes. The results were normalized to Ponceau S staining. Asterisks indicate statistically significant differences between control and cancer cells co-cultured with adipocytes. The significance level was set at *p* ≤ 0.05 (*), *p* ≤ 0.01 (**), *p* ≤ 0.0001 (****)

Furthermore, we checked the level of glucose transporters GLUT1 and GLUT3, which mediate basal glucose transport and favor the preservation of glycolytic energy metabolism when there is a limited supply of the substrate [32]. Quantitative RT-PCR and western blot analyses indicated increased mRNA (Fig. 6A) and protein (Fig. 6B) levels of GLUT1 in LS180/AD and HCT116/AD. Moreover, the transcript level of *GLUT3* was substantially elevated in LS180/AD and HCT116/AD (as a trend) in comparison to control cells (Fig. 6A). In addition to the increased expression, the western blot analysis revealed modification in the glycosylation pattern of GLUT1 glycoprotein in CRC co-cultured with adipocytes (Fig. 6B). Interestingly, the molecular weight of this transporter may be different for various CRC cell lines [33].

## Discussion

Among the many cellular elements, adipocytes are the main component of the colon cancer niche [34]. It is widely accepted that tumor cells might convert approximal adipocytes into CAAs. This may lead to changes in the tumor microenvironment that promote cancer progression [5]. Due to evidence showing the pro-tumorigenic effects of CAAs, this interplay between cancer cells and adipocytes in the tumor niche has gained interest. However, there is relatively little research on the interaction between adipocytes and CRC cells. Therefore, in this study, we focused on the mutual relationship between these cells.

To explore the bi-directional interactions between adipocytes and colorectal cancer cells, we decided to use the 3T3-L1 pre-adipocyte cell line as an experimental model. Notably, there is no perfect model for this type of research. After differentiation, human primary preadipocytes may better reflect in vivo conditions, but they have a limited number of divisions and are much more difficult to cultivate than 3T3-L1 cells. Moreover, they descend from individuals, so it is hard to obtain repeatable quantitative results and come to appropriate conclusions. Due to the use of mouse pre-adipocytes, which more efficiently multiply than human cells, it was possible to expand the number of colorectal cell lines and apply several functional tests (like proliferation and migration assays).

Other models that could be used in this type of research are mouse preadipocytes and mouse cancer cell lines. However, we believe that results obtained from their co-culture do not reflect mechanisms observed in human cells, so the results could not be directly applied to humans. Each of the mentioned models has its limitations, so based on available literature data, we decided to use the 3T3-L1 pre-adipocyte cell line. These cells are commonly employed in in vitro studies [18] and were previously employed as a model of co-cultured adipocytes with colon cancer cells [35]. After differentiation, 3T3-L1 pre-adipocytes present the gene expression profile and basal bioenergetics typical for white adipocytes [36], so they seem to be a good model.

As mentioned, under exposure to cancer cells, adipocytes can transform into cancer-associated adipocytes [7]. Zoico et al. revealed that during interaction with colon cancer cells, adipocytes obtained from 3T3-L1 cells can be reprogrammed into CAAs or dedifferentiated into “fibroblast-like cells” in vitro [35]. Similarly, we observed that all tested CRC cells converted adipocytes into cancer-associated adipocytes, which decreased lipid content and the expression of genes that are characteristic of adipocytes (including *PPARγ*, *resistin*, *adiponectin*, *FABP4*, *perilipin,* and *LIPE*). Downregulation of these genes’ expression in adipocytes exposed to various types of cancer cells has been described (mostly in breast cancer [37]), but in the case of CRC cells, they still remain poorly explored.

The hallmark of adipocytes’ transformation into CAAs is the inhibition of adipogenesis, which is a multi-step process where preadipocytes are converted into mature adipose cells. During this transformation, four phases are distinguished: growth arrest, early differentiation (called mitotic clonal expansion (MCE)), intermediate differentiation, and late differentiation. PPARγ and C/EBPα cooperatively act in the intermediate step and promote the induction of several adipocyte-specific genes: *FABP4*, *resistin*, *adiponectin*, *LIPE,* and *perilipin* in the terminal stage of differentiation [18, 37]. Moreover, phosphorylation of the Akt kinase induces preadipocyte differentiation, whereas its inactivation inhibits this process. The ERK kinase pathway is essential for MCE, so this signal transduction path must be turned off to allow for adipogenic differentiation [24]. The diminished level of phosphorylated Akt (pAkt) and increased activation of ERK observed in our research confirmed that adipocytes inhibited adipogenesis upon stimulation with CRC cells.

The dispersed lipid droplets and a reduced amount of lipids in CAAs that we detected may be also a consequence of lipolysis, which occurs upon colorectal cancer-cell exposure. In this process, triglycerides are hydrolyzed to FFAs and glycerol [35, 38]. Wen et al. observed that the level of glycerol secreted into the medium by adipocytes was increased in the presence of CRC cells, suggesting that cancer cells promoted lipolysis [39]. Additionally, it was revealed that STAT3 is a key regulator of lipolysis-driven oxidative metabolism in adipocytes. This protein is phosphorylated in a lipolysis-dependent manner in response to β-adrenergic activation and redirects fatty acids toward oxidation [25].

Interestingly, Lewis lung carcinoma cells secrete extracellular vesicles containing interleukin-6 that induced lipolysis in 3T3-L1 cells via the STAT3 pathway [40]. This intracellular signaling way could be also activated by leukemia inhibitory factor and contribute to cancer cachexia [41], which affects more than half of patients with colorectal cancer [42]. Another pathway involved in lipolysis might be the ERK pathway, which also takes part in the adipogenesis process. Activation of this kinase is an early signal for the reduction of the perilipin protein level [26], so our observation of enhanced phosphorylation of ERK may be connected with the decreased expression level of *perilipin*. This protein is located on the surface of lipid droplets and protects them from the access of lipases [43].

In our observations, when there was reduced expression of *perilipin* in CAAs, lipid droplets may be more exposed to lipases. We also observed reduced expression of *LIPE* and *FABP4* genes in CAAs in comparison to adipocytes, which was also described in types of cancer (such as breast cancer [44]) and might seem to be inconsequential. A smaller level of proteins encoded by these genes would be expected to decrease lipolysis in adipocytes. However, this finding suggests that cancer cells could regulate delipidation via mechanisms that are independent of altered expression of lipolytic genes [37].

Furthermore, decreased amounts of lipid droplets in CAAs can be partially explained by reduced expression of genes encoding enzymes involved in fat synthesis in these cells. For the first time, we noticed that adipocytes co-cultured with CRC cells had lower levels of mRNA of *SC4MOL* (which is implicated in cholesterol biosynthesis) and *OSBPL9* (which encodes an oxysterol-binding protein that belongs to a group of intracellular lipid receptors). In addition, we also found evidence that adipocytes co-cultured with cancer cells had a diminished expression level of *FADS1*, which encodes the delta-5 desaturase, one of the key enzymes in polyunsaturated fatty acid synthesis [45].

While CAAs had poorer fat content, all tested cancer cells presented increased amounts of lipid droplets after co-culture with adipocytes. Nieman et al. had similar observations when co-culturing colon cancer cells with primary adipocytes [46]. Moreover, increased content of lipid droplets was observed in tumor tissues from patients with colon cancer in comparison to adjacent normal tissue [47]. Cruz et al. broadly described the functions of lipid droplets in cancer cells, and in CRC, they may stimulate the proliferation and transformation of cells toward a more invasive phenotype [48]. This was also observed in our experiments, where LS180 and LoVo cells co-cultured with adipocytes had an increased proliferation rate and migration abilities in 2D and 3D conditions.

The increased proliferation abilities for LS180/AD and HCT116/AD coincide with results obtained from cell cycle analysis. These cells shifted from G0/G1 into G2/M phases, but we did not observe any differences between LoVo and LoVo/AD cell-cycle phase distributions. Similarly, Bergqvist et al. observed that the secretome of obesity-associated adipocytes stimulated proliferation of breast-cancer cells and shifted the cell cycle from G1 toward S and G2/ M phases [49].

Additionally, we verified the expression level of chosen cyclins and CDK1. We observed a substantially diminished expression level of *CCND1* in all tested CRC cells upon exposure to adipocytes. This cyclin has a peak of expression in the early G phase [28], so its reduction may indicate that cells pass into later phases of the cycle. Interestingly, in colon polyps, cyclin D1 is overexpressed in comparison to normal tissue and may be linked with cell cycle-promoting activity [50]. Some evidence has revealed that expression of *CCND1* correlates with poor survival in a variety of human malignancies, including CRC [51–53]. On the other hand, the reduction of CCND1 in human cancer cells may decrease the homologous recombination and cause higher sensitivity of cells to DNA damage. Conversely, overexpression of this cyclin might override cell-cycle arrest followed by DNA damage, leading to apoptosis [54].

Furthermore, LS180/AD and HCT116/AD cells had significantly elevated expression of *CCNA2, CCNB1* (which was observed as a trend for HCT116/AD), and *CDK1* genes in comparison to the control. This probably explains the accumulation of cells in the G2/M phases. Low levels of cyclin A2 first occur at the G1/S boundary. The amount then rises steadily to the highest expression at the end of the S phase, when cells begin to replicate DNA and do not decline until cyclin A is degraded by ubiquitin-mediated proteolysis in the late G2 phase. Then, B-type cyclins (the M cyclins) are actively synthesized [28].

The level of these cyclins and CDKs were already investigated in CRC tissues. The mRNA and protein levels of cyclin A2 and cyclin B1 were increased in a tumor compared to normal samples and are promising biomarkers for the diagnosis and prognosis for CRC-suffering patients [52, 55, 56]. Also, the upregulation of CDK1 gene expression in colorectal compared to healthy tissue was observed in other studies [57]. Moreover, Li et al. demonstrated that *CCNB1* not only promotes malignant phenotypes by stimulation of the proliferation of cancer cells, but may also support cell invasion via the activation of epithelial-to-mesenchymal transition [56]. Despite the role of cyclins and CDKs in promoting cell growth and division, their level was not found to be excessive in cancer upon exposure to adipocytes. Lore et al. noticed that the secretome of cancer-associated adipose tissue stimulated the proliferation of breast-cancer cells and increased the protein level of cyclin A and cyclin E in comparison to the control [58].

CRC cells exposed to adipocytes need much energy for increased proliferation and migration, which they may derive from fatty acids stored in lipid droplets, among other sources. Accumulation of these structures may arise in cells through different mechanisms, including increased lipid uptake and de novo synthesis [48]. Although in normal adult tissues, the level of de novo synthesis of fatty acids is low [59], carcinogenesis is associated with increased demand for fatty acids [60]. Moreover, Reinfeld et al. demonstrated that obesity stimulated colorectal and renal cancer cells to adopt a lipid-based metabolism, while TME cells adapt to glucose [61].

During lipogenesis, FASN takes part in the assembly of malonyl-CoA into long fatty acid chains [62]. We noted that its level slightly increased in HCT116 and LoVo cells co-cultured with adipocytes. It was shown that the upregulation of enzymes involved in lipid biosynthesis correlates with tumor growth [59]. Higher activity of FASN was observed in breast-cancer cells [63]. Elevated fatty acid production could be a response to the high metabolic demand of cancer cells [7], which synthesize most fatty acids de novo [64]. Enhanced lipogenesis is a hallmark of many aggressive malignancies. The literature reports that in aggressive cancers, uptake of FFAs from their niche occurs only when endogenous lipogenesis becomes insufficient [9].

It is thought-provoking that adipocytes led to decreased the FASN level in LS180 cells. These cells may possibly derive energy from mainly glucose metabolism because strong overexpression of GLUT1 and GLUT3 transporters in LS180/AD cells was detected. The level of GLUT1 was also increased in HCT116 cells (based on mRNA and protein levels) and LoVo cells (based on only the mRNA level) that were co-cultured with adipocytes. This may indicate that CRC cells amplified the influx of glucose, which is an essential energy source for cells.

Both GLUT1 and GLUT3 transporters are responsible for basal glucose uptake in cancer cells [33] and have identical substrate coordination, but GLUT3 has a higher glucose affinity than GLUT1 [65]. They are overexpressed in many types of tumors, which is associated with malignancy [32, 66]. Interestingly, Hauptmann et al. observed the highest expression of GLUT1 in the most slowly proliferating colon cell lines. This may suggest that high glucose consumption is required for the synthesis of various products as a source of energy or structural components rather than for proliferation. They also indicate that GLUT1 activity depends on structural modifications, including glycosylation. This transporter undergoes N-glycosylation on asparagine 45 [67], which plays an important role in maintaining a fully active structure of the protein but does not interfere with its intracellular targeting [68].

Thus, even though cells express this transporter on a comparable level, they may uptake glucose in different amounts because of distinctions in glycosylation patterns [33]. In addition, enormous glucose consumption by cancer cells under influence by adipocytes may be connected with the Warburg effect [59]. Despite the low efficiency of aerobic glycolysis, it is a fast way to synthesize ATP and has benefits for cancers localized in a microenvironment with limited availability of glucose or when tumors compete with stromal cells for this energy source [69].

It was demonstrated that lactate released by TME may be an additional substrate of energy for cancer cells [70]. Our results showed increased release of lactate by adipocytes co-cultured with CRC cells compared with the mono-cultured control. Moreover, we noticed the overexpression of *monocarboxylate transporter 1* in AD/HCT116 and AD/LoVo and overexpression of *Glut1* in AD/LS180. The transport of lactate into the tumor niche by MCT1 is coupled to the cotransport of H + and causes acidification of the TME [59]. Moreover, NHE1, which was overexpressed in observations, takes part in the maintenance of intracellular pH homeostasis via extrusion of H^+^ ions generated in the cytosol [27], thereby causing further acidification of the microenvironment. It is possible that CRC cells could stimulate the intake of glucose by adipocytes, which metabolize this carbohydrate into lactate and secrete it via MCT1 into the medium. However, we do not have enough evidence to confirm this hypothesis, so this area requires further research.

Nevertheless, it was demonstrated that lactate produced by stromal cells can be consumed by tumors and become one of the energy sources for CRC cell migration. Liu et al. showed improved overgrowth of the scratch and invasion abilities of LoVo cells after lactate treatment [71]. This is in agreement with our observation that adipocytes stimulated migration and invasion of LoVo and LS180 cells. Additionally, it was shown that the high level of lactate in the TME is positively associated with distant metastasis and a poor prognosis in various types of cancer, including CRC [71].

## Conclusions

In summary, our studies showed that colorectal cancer cells drove the reprogramming of adipocytes into CRC-associated adipocytes in vitro. We found that the delipidation observed in adipocytes upon exposure to CRC cells may be the result of inhibition of adipogenesis, increased lipolysis, and the reduction of expression of genes involved in lipid metabolism. For the first time, our study showed lower expression of genes involved in lipid synthesis (*OSBPL9, SC4MOL*, *FADS1*) in adipocytes co-cultured with CRC cells compared with those from mono-culture. On the other hand, adipocytes reprogrammed by colorectal cancer cells into CAAs might support CRC cells in various ways. They secrete metabolites like lactate, which may be energy substrates for tumor cells. As a result, culture with CAAs promoted lipid accumulation in all tested colorectal cancer cells.

**Fig. 7:**
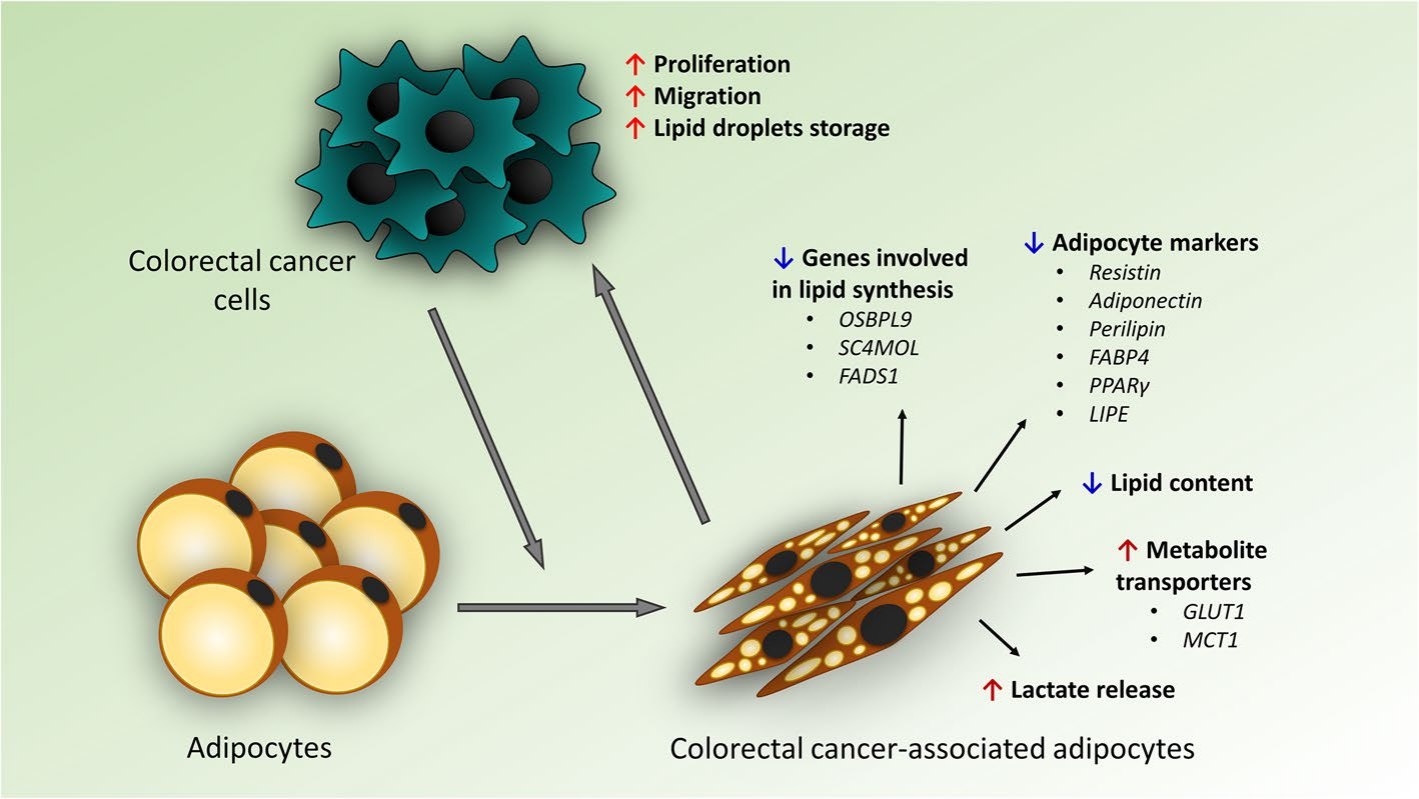
Cross-talk between colorectal cancer cells and adipocytes. Abbreviations: SC4MOL: sterol-C4-methyl oxidase-like protein, OSBPL9: oxysterol-binding protein-like 9, FADS1: fatty acid desaturase 1, MCT1: monocarboxylate transporter 1, NHE1: sodium–hydrogen antiporter 1, GLUT1: glucose transporter 1, PPARγ: peroxisome proliferator-activated receptor γ, MCT1: monocarboxylate transporter 1, LIPE: hormone-sensitive lipase, FABP4: fatty acid binding protein 4

In addition, we noted an increased proliferation rate that switched the distribution of cell cycle phases from G0/G1 into G2/M and improved migration and invasion abilities of CRC cells co-cultured with adipocytes. The research highlighted the complex bi-directional relationship between adipocytes and colorectal cancer cells (Fig. 7). Understanding this process could contribute to the improvement of knowledge on the role of the tumor microenvironment in the progression and mechanisms of colorectal cancer linking CRC with obesity.

## Data Availability

The datasets during and/or analyzed during the current study are available from the corresponding author on reasonable request.

## Abbreviations

CAAs: Cancer-associated adipocytes
CRC: Colorectal cancer
TME: Tumor microenvironment
ECM: Extracellular matrix
AT: Adipose tissue
FFAs: Free fatty acids
PPARγ: Peroxisome proliferator-activated receptor γ
c/EBPα: CCAAT/enhancer-binding protein α
FABP4: Fatty acid binding protein 4
LIPE: Hormone-sensitive lipase
BCS: Bovine calf serum
FBS: Fetal bovine serum
EeF2: Eukaryotic translation elongation factor 2
HPRT1: Hypoxanthine phosphoribosyltransferase 1
SC4MOL: Sterol-C4-methyl oxidase-like protein
OSBPL9: Oxysterol-binding protein-like 9
FADS1: Fatty acid desaturase 1
MCT1: Monocarboxylate transporter 1
NHE1: Sodium–hydrogen antiporter 1
GLUT1: Glucose transporter 1
GLUT3: Glucose transporter 3
CCND1: Cyclin D1
CCNA2: Cyclin A2
CCNB1: Cyclin B1
CDK1: Cyclin-dependent kinase 1
Akt: Protein kinase B
pAkt: Phosphorylated protein kinase B
ERK: Extracellular signal-regulated kinase
pERK: Phosphorylated extracellular signal-regulated kinase
STAT3: Signal transducer and activator of transcription 3
pSTAT3: Phosphorylated signal transducer and activator of transcription 3
